# The COVID-19 Pandemic as a Public Health Teacher—the Lessons We Must Learn

**DOI:** 10.3389/phrs.2024.1607232

**Published:** 2024-05-07

**Authors:** Sofia Lombatti, Avi Magid, Nadav Davidovitch, John Middleton, Mohamud Sheek-Hussein, Henrique Lopes, Natia Skhvitaridze, Hazem Agha, Daniel Lopez-Acuña, José Martínez Olmos, Ariane Bauernfeind, Vladimir Prikazsky, Nora Vesela, Alena Petrakova, Gaetano Pierpaolo Privitera, Jean Philippe Naboulet, Lore Leighton, Robert Otok, John Reid

**Affiliations:** ^1^ Association of Schools of Public Health in the European Region (ASPHER), Brussels, Belgium; ^2^ Population Health Sciences, University College London, London, United Kingdom; ^3^ School of Public Health, Ben Gurion University of the Negev, Beer-Sheva, Israel; ^4^ Rambam Health Care Campus, Haifa, Israel; ^5^ Faculty of Education, Health and Wellbeing, University of Wolverhampton, Wolverhampton, United Kingdom; ^6^ Institute of Public Health, College of Medicine, United Arab Emirates University, Al Ain, United Arab Emirates; ^7^ School of Public Health, Loma Linda University, Loma Linda, CA, United States; ^8^ NOVA Center for Global Health, NOVA University Lisbon, Lisbon, Portugal; ^9^ School of Health Sciences, University of Georgia, Tbilisi, Georgia; ^10^ Faculty of Public Health, Al-Quds University, Jerusalem, Palestine; ^11^ Andalusian School of Public Health, Granada, Spain; ^12^ Faculty of Medical Studies, Technical University of Liberec, Liberec, Czechia; ^13^ Faculty of Health Sciences, Palacký University Olomouc, Olomouc, Czechia; ^14^ Faculty of Medicine and Surgery (Facoltà di Medicina e Chirurgia), University of Pisa, Pisa, Italy; ^15^ Département METIS—EHESP, Rennes, France; ^16^ School of Allied and Public Health, University of Chester, Chester, United Kingdom

**Keywords:** COVID-19, pandemic, lessons learned, recommendations, ASPHER

**Statement of the Association of Schools of Public Health in the European Region (ASPHER)**

## Learning From the Pandemic

Since 5th May 2023 COVID-19 is not considered a Public Health Emergency of International Concern (PHEIC), rather “*an established and ongoing health issue*” [1]. This was a moment to celebrate achievements, like the dedication of healthcare workers but also to reflect on the mistakes made [1]. Many COVID-19 deaths are still reported to the World Health Organization (WHO) and reminds us to learn from the past and take advantage of windows of opportunity before the next large-scale global health emergency. The Association of Schools of Public Health in the European Region (ASPHER)’s 20 key lessons learned and recommendations, draw from three broad influences: international reports, ASPHER COVID-19 Task Force publications, and country perspectives.

## Seven Helpful International Reports on Lessons Learned

i) **The United Nations General Assembly 2023 High Level Meeting (HLM) on Pandemic Prevention, Preparedness, and Response (PPPR)** led to the Political Declaration by leaders [2]. This calls for future “*timely, urgent and continued leadership, global solidarity (…) to strengthen pandemic prevention, preparedness and response, and fully address the direct and indirect consequences of future pandemics*” [2]. Their 75 lessons include health inequalities which were severely exacerbated, the need to build and maintain global solidarity and trust, prioritise equity, and to maximise political will to build upon the lessons learned and best practices from the COVID-19 pandemic [2].
ii) **European Centre for Disease Control and Prevention (ECDC)—Technical Report (2023) on “Lessons Learned from COVID-19 Pandemic”** four lesson areas were identified to strengthen preparedness and response plans: *Investment in the public health workforce, preparing for the next public health crisis, risk communication and community engagement, and collection and analysis of data and evidence* [3]. Building relationships of trust between governments and their citizens is essential for an effective response to the next Public Health (PH) emergency, using transparent evidence-informed decision-making, which may increase community engagement [3].
iii) **Long Term COVID-19 Disease Management”—World Health Organization (2023)** [4]. Five areas need to be strengthened: *collaborative surveillance, community protection, safe and scalable care, access to countermeasures, and emergency coordination* [4].
iv) **The Lancet Commission on “Lessons for the Future”** echoed many global perspectives in calling for a 10-year global strategy [5]. These included broad support for global collaborations, WHO reform with extra powers and budgets, advocating for science based decision-making and strengthening global PH workforces. Specific lessons include a “*dual track*” for preventing emerging infections, including seeking to prevent “*natural spillovers*” and increasing biosafety and biosecurity in pathogen research [5].
v) **Expert Consensus Panel Report.** They highlighted many issues and recommendations and the levels of agreement achieved. Six broad cross-cutting themes were identified highlighting issues - such as vaccines-plus approaches, building public trust and being community centred [6]. The lead author, with others, later highlighted that current international treaties and health regulations, and accountability and enforcement mechanisms, should be improved [7].
vi) **The Independent Panel for Pandemic Preparedness and Response (IPPPR) report of 2021 (*COVID-19 Make it the last Pandemic*)** highlighted issues such as the need for rigorous and systematic use of non-pharmaceutical countermeasures and high vaccine coverage and failure to learn sufficiently from SARS and other previous infectious disease emergencies [8]. The IPPPR 2023 “Roadmap” recommends better use of the outbreak and pandemic cycle, using the interpandemic phase for stronger preparedness. Six broad “*essential functions for pandemic preparedness and response*” were identified, including a transformed “ecosystem for rapid and equitable access” to medical countermeasures [9].
vii) **The European Observatory on Health Systems and Policies (2023)** covered 20 strategies to reallocate resources and not leave anyone behind [10]. Key areas for future improvement were: “*leading and governing the COVID-19 response, financing COVID-19 services, mobilizing and supporting the health workforce and strengthening public health interventions”* [10].

Often recommendations are broad, with undefined target groups, and the extent to which countries implement them difficult to assess. EU Member States have different priorities, budgets and possibilities to address future emergency preparedness. No “*one-size fits all*” exist during PH emergencies [10], hence operational plans need to be translated into different health ecosystems.

Identifying what pandemic strategies worked, and learning from success, should offer solutions for stronger evidence-based emergency preparedness plans and capacities and capabilities across Europe and globally. Cited reports above underline the importance to strengthen healthcare workforces, build PH capacity, implement economic/financial measures, create better IT and data-sharing systems, and foster multidisciplinary coordination across different sectors.

## The Role of ASPHER COVID-19 Task Force

ASPHER convened a COVID-19 Task Force (TF) early in 2020 to facilitate networking among PH professionals to help them face the emergency and coordinate ASPHER’s actions across Europe [11]. This expert forum shared latest information, presented and reviewed evidence on many pandemic aspects, including epidemiological, technical, societal, and political dimensions. Collaborating with European and National Health Authorities, and non-governmental organisations, the TF helped accelerate coordination of policy responses.

ASPHER’s TF published over 30 articles, including on face masks, testing, tracking, vaccination, health inequalities, safe schools, advocacy for wider social protection and global vaccine equity. ASPHER has a pivotal role in shaping future plans, for our own academic PH responsibilities, but also in advising our partner European institutions and each country.

ASPHER’s previous Reports included: “Covid-19 Pandemic Waves Surveillance”; “The Need for Vaccine Internationalism”; “Handbook on Basic Epidemiological Terms” which supported journalists and non-specialists; ASPHER’s first inequalities statement “How the Pandemic is Amplifying Health Inequalities in Europe”; Handbooks of basic terms for health inequalities and for phone-based apps for contact tracing; and a statement on the “Reopening of Schools of Public Health—Rapid Review Survey” highlighted how European public health schools planned to reopen campuses.

## Country Level Perspectives

ASPHER’s TF identified country-level “lessons learned” perspective examples across Europe. We recognised specific lessons for countries or regions, related to their populations’ health needs, cultures and vulnerabilities, strength of their PH workforces, and varying access to universal healthcare. Lessons can be learned from localised PH systems given different severe pressures localities initially experienced, such as Northern Italy or lower vaccine population coverage in parts of Eastern Europe.

## Conclusion

Lessons from the COVID-19 pandemic must be acted upon. Mistakes, including inevitable “public health errors” [12], were made but strong progress was made in epidemiology, healthcare and health sciences, and wider academic and other scientific fields.

The pandemic highlighted the urgency of developing harmonised PH and competencies for epidemiology and other areas [13], and also better global governance [14]. The ECDC-ASPHER Competency Framework for applied infectious disease epidemiology, exemplifies harmonised approaches to investigating and controlling infectious diseases and capacity building of PH workforces across Europe [15].

The ASPHER COVID-19 Task Force, now “Public Health Emergencies Task Force” will continue work to improve emergency preparedness. This will help harmonise new approaches post-pandemic, for PH to be better equipped and more resilient in future emergencies. We identified 20 priority lessons/themes below, to guide ASPHER.

## Twenty Priority Lessons Learned for ASPHER From the Pandemic

1. Ensure ASPHER’s independent and trusted voice.
2. Further our inter-country collaborative culture and mutual support.
3. Support global collaboration and governance for pandemics and other emergencies.
4. Support cross-border cooperation and consistency.
5. Harness new ICT and remote working skills.
6. Continue horizon scanning and alertness for future threats.
7. Promote timely, well-resourced and comprehensive Epidemiological Reporting systems.
8. Advocate for reductions in health inequalities and protecting vulnerable population groups before and during future pandemics.
9. Promote universal healthcare systems and recognition of the care dividend.
10. Support more equitable vaccination systems and combat vaccine hesitancy.
11. Strengthen capacity and combat erosion of PH workforces, particularly focussing on PH infrastructure for improving discharge of essential PH functions at local, regional and national level.
12. Ensure PH competencies for future disasters and pandemics.
13. Strengthen evidence-informed PH practice, including early Health Impact Assessments.
14. Prepare pandemic and epidemic recovery plans to restore reduced health status lost, including life expectancy.
15. Promote plans to combat syndemic deaths and illness during crises.
16. Promote resilient and credible robust governmental guidance from expert groups to support future decision-making on public health policy.
17. Strengthen the role of PH with health authorities and political decision-makers in pandemic crises situations.
18. Improve preparedness in health crisis communications among PH professionals.
19. Support ethical and humanitarian concerns of PH professionals, including financial stability for vulnerable populations.
20. Share knowledge, skills and resources for medical and health technologies rapidly, widely and equitably.

## Editorial Note

The authors are responsible and liable for the contents of this Society Statement. Society Statements in PHR are checked for clarity and approved by an Editor-in-Chief. Society Statements are not externally peer reviewed.
